# Precision surgery for endometriosis: preventing chronic pelvic pain in patients with higher pre-operative pain scores and in patients of advanced age

**DOI:** 10.1007/s00404-025-07996-7

**Published:** 2025-03-26

**Authors:** Davit Bokhua, Angela Kather, Anna Kaufmann, Evangelia Polychronaki, Valentina Auletta, Ingo B. Runnebaum

**Affiliations:** 1 Department of Gynaecology and Reproductive Medicine, Jena University Hospital, Friedrich Schiller University Jena, Am Klinikum 1, 07747 Jena, Germany; 2 Zentrum für Alternsforschung Jena – Aging Research Center Jena, Jena, Germany; 3 RU21 GmbH, Botzstraße 3, 07743 Jena, Germany

**Keywords:** Endometriosis, Surgery, Non-responder, Chronic pelvic pain, Fertility, Quality of life

## Abstract

**Objective:**

Symptom relief can be achieved for many patients with endometriosis by tailored individual treatment. However, therapy resistance is observed in some patients. This study surveyed patient-reported long-term outcomes after laparoscopic endometriosis surgery and evaluated potential pre-operative predictors for insufficient symptom control.

**Methods:**

This retrospective study included patients with complete surgical endometriosis resection treated between 2013–2016 at the Department of Gynaecology and Reproductive Medicine, Jena University Hospital. Our 2020 survey gathered socio-demographic, reproductive, symptom-related, and subjective general condition data from 122 patients. Overall pain intensity was assessed using a numeric rating scale (NRS) from zero (no pain) to 100 (highest imaginable pain). Clinical records provided additional information.

**Results:**

Median time between surgery and interview was 6 years. Postoperatively, the proportion of patients reporting symptoms was considerably reduced (menstrual pain 32.0% vs. 85.2%, chronic pelvic pain [CPP] 40.2% vs. 67.2%, dyspareunia 34.4% vs. 59.8%, hypermenorrhea 17.2% vs. 49.2%; p < 0.001). The majority of respondents (70%) reported improved subjective general condition. Mean NRS Score significantly decreased from 77.2 to 26.6 (p < 0.001). Among pre-operatively infertile women, 45.2% reported successful pregnancies. However, 20–30% of patients did not respond to therapy in one of the analyzed domains. Multivariate logistic regression identified CPP as a strong predictor for failure in permanent pain reduction (OR 5.544, 95% CI 1.338–22.965, p = 0.018) and risk for reoperation (OR 5.191, 95% CI 1.100-24.501, p = 0.038). Higher pre-operative NRS scores and increasing age were associated with better long-term pain relief.

**Conclusion:**

Patients with higher pre-operative pain scores and patients of advanced age benefit significantly from precision surgery, experiencing sustained symptom relief and improved subjective general condition. However, younger patients with CPP and moderate pre-operative pain intensity showed a higher risk for therapy resistance and require multimodal treatment strategies.

**Supplementary Information:**

The online version contains supplementary material available at 10.1007/s00404-025-07996-7.

## What does this study add to the clinical work

**Table:** 

Patients with higher pre-operative pain scores and patients of advanced age benefit significantly from precision surgery, experiencing sustained symptom relief and improved subjective general condition. However, younger patients with chronic pelvic pain and moderate pre-operative pain intensity showed a higher risk for therapy resistance and require multimodal treatment strategies.	

## Introduction

Endometriosis is a benign, estrogen-dependent disease associated with chronic pelvic pain (CPP) and infertility [1]. The prevalence of endometriosis in women of reproductive age is 6–10%, in patients with infertility 30–50%, and in women with CPP 50% [2]. In Germany, approximately 40,000 new cases are reported annually [3]. The economic impact of endometriosis, for example, in Germany and the UK, is significant, reflecting substantial costs in healthcare and productivity losses. Endometriosis incurs an annual economic burden of approximately €12.5 billion in Germany and £7.6 billion in the UK [4], emphasizing the need for effective management strategies.

Though the etiology and pathogenesis of endometriosis have been extensively studied, a definitive answer has yet to be found. According to various theories, endometriosis is a multifactorial disease, in the formation of which participate genetic, neurologic, inflammatory, and immunologic factors [5, 6].

Clinical symptoms characteristic of the disease are dysmenorrhea, CPP, dyspareunia, infertility, dyschezia, and dysuria. Nonspecific symptoms include bowel and bladder dysfunction, back or limb pain and headache, nausea, chronic fatigue. Type of symptoms correlates with location of endometriosis [7]. In 20–25% of cases, the disease is progressing asymptomatically [8]. Due to the heterogeneity of symptoms, the disease is often diagnosed with a 8–10-year delay [9]. Endometriosis represents one of the most prevalent reasons for infertility [10–12] and can also impede assisted reproductive technology [13].

In addition to physical complaints, in endometriosis often there are reported neuro-psychic disorders (depression, asthenia, fatigue, and general weakness), which negatively affect the quality of life and cover many aspects of women’s lives, including the social sphere, mental health, sexual life and working capacity [14]. Endometriosis violates the main indicators of quality of life, such as social role performance, adaptation, mental well-being and functioning in social groups [15].

Previous studies have mainly focused on how dysmenorrhea, infertility, and pelvic pain affect quality of life [16, 17]. Pessoa et al. found that symptoms that have a particularly negative impact on quality of life include menstrual pain, pelvic pain, dysmenorrhea, and dyspareunia [18].

Chronic pelvic pain (CPP) is a major cause of morbidity in patients with endometriosis. Endometriosis is present in approximately 40–87% of patients with CPP, making it the most commonly identified cause of CPP. The extent of endometriosis-related CPP (endo-CPP) varies widely and is a multifactorial process that has nociceptive, neuropathic and nociplastic features [19]. A chronic inflammatory response induced by endometriosis leads to the development of CPP and, as a result, has severe consequences in terms of alteration of brain structure and reduction of gray-matter volume in voxel-based brain morphometry and fMRIs, as shown in publications by As-Sanie et al. [20, 21]. Among others, endo-CPP is often associated with myofascial dysfunction and sensitization beyond the pelvic region, which can be triggered or maintained by persistent pelvic floor spasms [22–24]. The complex pathophysiology of CPP requires a transdisciplinary treatment approach [25].

Endometriosis treatment focuses on symptom management, as no causal therapy exists. Individualized treatment plans must balance conservative and surgical approaches based on symptomatology, reproductive goals, and organ involvement [26]. No single treatment modality has demonstrated superior efficacy for pain relief [27]. Thus, guidelines recommend the least invasive option [26, 28]. Advances in imaging facilitate noninvasive diagnosis in most cases [29], minimizing unnecessary surgery. Consequently, patients should not undergo surgery without a thorough and reliable diagnosis and hormonal therapy is now widely applied as first line treatment. Primary surgical resection remains common, particularly for patients desiring immediate conception [28, 30], those avoiding long-term hormonal therapy due to side effects [31], or cases involving organ dysfunction or extensive tissue destruction.

Laparoscopic surgery offers best perioperative results with less complications and faster recovery [32]. Furthermore, it has been demonstrated as the most cost-effective approach for providers, patients, and healthcare systems. It is crucial to emphasize that surgical teams lacking adequate training and expertise in complex laparoscopic endometriosis surgery should prioritize patient safety and refer them to specialized centers rather than proceeding with laparotomy or minilaparotomy.

Pain improvement is not sufficiently achieved after surgery in approximately one quarter of patients [33, 34]. This can be improved by applying postoperative hormonal therapy [35]. However, 13–16% (Dienogest) respectively 24.5% (MPA = medroxyprogesterone acetate) of patients still experience recurrence of endometriomas and/or symptoms [35–37]. Options for treatment of recurrent endometriosis are the same as in the primary situation with individual consideration of medical (hormonal) therapy and/or surgery [28]. Surgery in the recurrent situation appears to have the same efficiency and limitations as primary surgery [38]. However, the body of evidence for this conclusion is rather small.

A recent systematic review identified only five studies which investigated prognostic factors for successful pain reduction after endometriosis surgery [39]. Our retrospective study aimed to investigate patient-focused long-term outcome of surgery for endometriosis at the Gynaecology Department of the University Hospital of Jena and to contribute to the identification of predictive factors for pain improvement, risk of reoperation and restoration of fertility. Furthermore, we aimed to evaluate our concept of precision surgery regarding patient reported long-term symptom relief and health improvement. Precision surgery in endometriosis involved the complete excision of endometriotic tissue while preserving organ integrity and sparing nerves. This approach was facilitated by advanced visualization techniques, including 4K high-resolution laparoscopy and 3D technology, including robotic-assisted surgery, to enhance surgical precision and optimize outcomes.

In this study, we also examined the aspect of age in relation to endometriosis outcomes, specifically investigating how age influences long-term pain relief and overall treatment efficacy following surgical intervention.

## Material and methods

### Patients and study design

A single-center, retrospective study was conducted in 2020 at the Gynaecology and Reproductive Department of Jena University Hospital, a Level 3 certified interdisciplinary endometriosis center recognized by the Endometriosis Research Foundation and accredited by the German AGE (Association for Gynaecologic and Obstetric Endoscopy e.V.). As a tertiary referral hospital, our center serves a population of approximately 2-million people. The study cohort comprised patients referred to our center with a strong suspicion of endometriosis as the underlying cause of their pain or infertility, following prior inconclusive diagnostic evaluations. Diagnostic laparoscopy with complete excision of visible endometriotic lesions was performed when imaging techniques were insufficient or when patients expressed a strong preference for surgery to obtain a definitive diagnosis.

Our center employs a transdisciplinary model in collaboration with the radiology department, psychologic services, and a specialized pain unit: All patients were first evaluated at our specialized endometriosis outpatient clinic. In cases with suspected advanced-stage disease, MRI was performed and reviewed in biweekly interdisciplinary radiology sessions. While we lack specific data on pre- and postoperative physical therapy, all patients typically received physical rehabilitation during their postoperative hospital stay. Perioperatively, all patients received continuous psychologic support before and after surgery. Patients were systematically assessed for chronic pain syndromes, including generalized pain conditions and chronic overlapping pain conditions (COPC). If chronic pain was suspected, patients were referred to the pain unit for further assessment and interdisciplinary decision-making regarding their treatment strategy. This included discussions on whether to proceed with surgical intervention or alternative multimodal pain management strategies (e.g., systemic pharmacologic and psychologic pain therapy).

The criteria for inclusion in the study were histologically confirmed diagnosis of endometriosis, complete surgical resection of all visible endometriosis in the entire abdomen, and availability of intra- and postoperative documents. All patients were offered psychologic support throughout their entire stay at the hospital, with female psychologists trained in endometriosis care included in the daily ward rounds. Complete surgical resection was achieved through minimally invasive laparoscopic surgery standardized for endometriosis, which involved thorough systematic inspection and treatment of the entire peritoneal cavity (see surgery paragraph below). The procedure utilized the Olympus high-resolution 4K system, providing enhanced magnification and visualization capabilities to ensure meticulous resection of endometriotic lesions. Sporadically, the open abdominal approach ex g. as minilaparotomy was chosen because of difficult to reach endometrioma locations or particular bowel involvement. In the period of years 2013–2016 a total of 401 cases were identified. Eighteen cases represented repeated surgery of the same patient, resulting in a total number of included patients of n = 383. At the next stage, patients were informed in writing about the survey and voluntary participation in the study, accompanied by the approval of the ethics committee (No. 2020–1817-Bef). Between September 2020 and December 2020, 122 patients were interviewed by phone, 44 refused and 217 could not be contacted.

The questionnaire was developed by the authors and included the following items: socio-demographic data, subjective complaints, frequency of typical symptoms of endometriosis before and after surgery, severity of pain and subjective general condition before and after surgery, reoperation and fertility (pregnancy, live birth, abortion, complications and need of reproductive therapy). For evaluation of overall pain intensity, patients were asked to score their pain sensation using a numeric rating scale (NRS) ranging from zero (no pain) to 100 (highest imaginable pain).

Assessment of change in subjective general condition was limited to one question (“Do you think your quality of life has improved after surgery?”) with only binary answer categories (“yes” resp. “no”). In addition, subjective general condition at the time of interview was queried using the Short-Form-12 (SF–12) health survey [40], including scores for physical and mental health. Data were complemented with information from medical documentation.

### Surgery

In the context of endometriosis, precision surgery aimed to achieve complete excision of endometriotic tissue to alleviate the specific symptoms and minimize the risk of recurrence while preserving organs and sparing nerves by separating and distancing them from the peritoneum and fibrotic lesions. This approach sought to prevent long-term complications like CPP or infertility. The visualization systems allowed surgeries to be highly targeted and tailored to individual patients’ needs using minimally invasive techniques such as 4K high-resolution laparoscopy or 3D technology including robotic surgery. These methods allowed for the differentiation of tissues and precise identification and complete removal of endometriotic lesions sparing healthy tissue.

Particular attention was given to the inferior hypogastric plexus formed in part by pelvic splanchnic nerves, sacral splanchnic nerves and the superior hypogastric plexus plus visceral afferent fibers. Like procedures in radical cervical cancer surgery the location and characteristics of these associated nerves were carefully considered. Special focus was placed on the anatomic relationship between the hypogastric nerve and the uterosacral ligament to prevent postoperative morbidity during surgery for deep infiltrative endometriosis involving the uterosacral ligament.

The surgical plan was customized based on each patient’s specific condition including the extent and location of endometriosis-associated symptoms and overall health status. This personalization considered the patient’s fertility goals and the presence of other medical conditions. The technical expertise required a high level of surgical skill and experience particularly in complex cases where precision was crucial. These surgeries (including multivisceral excisions including bowel surgeries) were performed by the gynecological oncologists IBR and team, particularly skilled in minimally invasive radical surgery.

### Statistical analysis

Statistical analysis was performed using IBM SPSS version 27. Categorial variables were analyzed using Chi-squared or Fisher’s exact test, as appropriate. Differences between pre- and postoperative mean values of the NRS Score were tested using Wilcoxon signed rank-test because Shapiro–Wilk-Test indicated no normal distribution. Impact of patient and disease characteristics on postoperative NRS Score was analyzed with univariate ANCOVA analysis. Risk for therapy resistance was investigated using univariate and multivariate logistic regression. A p value ≤ 0.05 was considered statistically significant.

## Results

One hundred and twenty-two patients completed the questionnaire. Median time between surgery and interview was 6 years (range 4.3–7.8 years). Average age was 35.7 years (range 20.1–57.9), and mean body mass index (BMI) was 25.4 kg/m^2^ (range 17.2–41.6). Patients’ characteristics are presented in Table 1. The majority of patients underwent laparoscopic surgery. In 5.7% (n = 7) of cases, the open abdominal approach was chosen or laparoscopy had to be converted, mainly because of endometriosis lesions at difficult to reach locations. Sole endometriosis resection and adhesiolysis was conducted in 77.9% (n = 95) of patients. Adnexectomy had to be performed in 13 patients and eight patients underwent hysterectomy. In 12 patients (9.8%), endometriosis lesions were identified in the bowel wall (rectum and/or sigmoid). Six of these patients required bowel segment resection with anastomosis, while two underwent full-thickness partial bowel wall resection using a tangential stapler approach. In four cases, a superficial resection involving only the serosa was possible.

**Table 1:** Basic characteristics of patients

Variable	n	Percent
General history (n =122)		
Non-smokers	56	45.9
Mental work	42	34.4
Hard physical work	39	32.0
Engaged in sports	72	59.0
Stable family life	105	86.1
Pain in surgical scars	15	12.6
Grade of the disease (rASRM-Score) (n =113)		
Grade I	36	31.9
Grade II	21	18.6
Grade III	24	21.2
Grade IV	32	28.3

Regarding postoperative medication, 51.6% of patients (n = 63) underwent hormonal therapy. Most of them (74.6%, n = 47) were treated with pure progestins, including two with progesterone releasing intrauterine device. Combined oral contraceptives were used by 16 patients. No information on postoperative hormonal treatment could be obtained in ten cases and two women received estradiol alone for HRT after bilateral adnexectomy. Based on available data, progestins were predominantly prescribed to patients with high pre-operative NRS (mean pre-operative NRS = 56.9 in patients without hormonal therapy vs. 80.5 in patients with postoperative progestin therapy, Supplementary Figure 1). Twenty eight (60%) of the 47 patients without postoperative hormonal treatment had immediate wish to conceive. For the remaining 19 patients it was retrospectively not possible to decipher, why they did not receive hormonal medication. However, the number could be explained by the fact, that our center is located in a region of Germany with a high rate of women being skeptical about hormonal medication.

### Symptom control

**Fig. 1:**
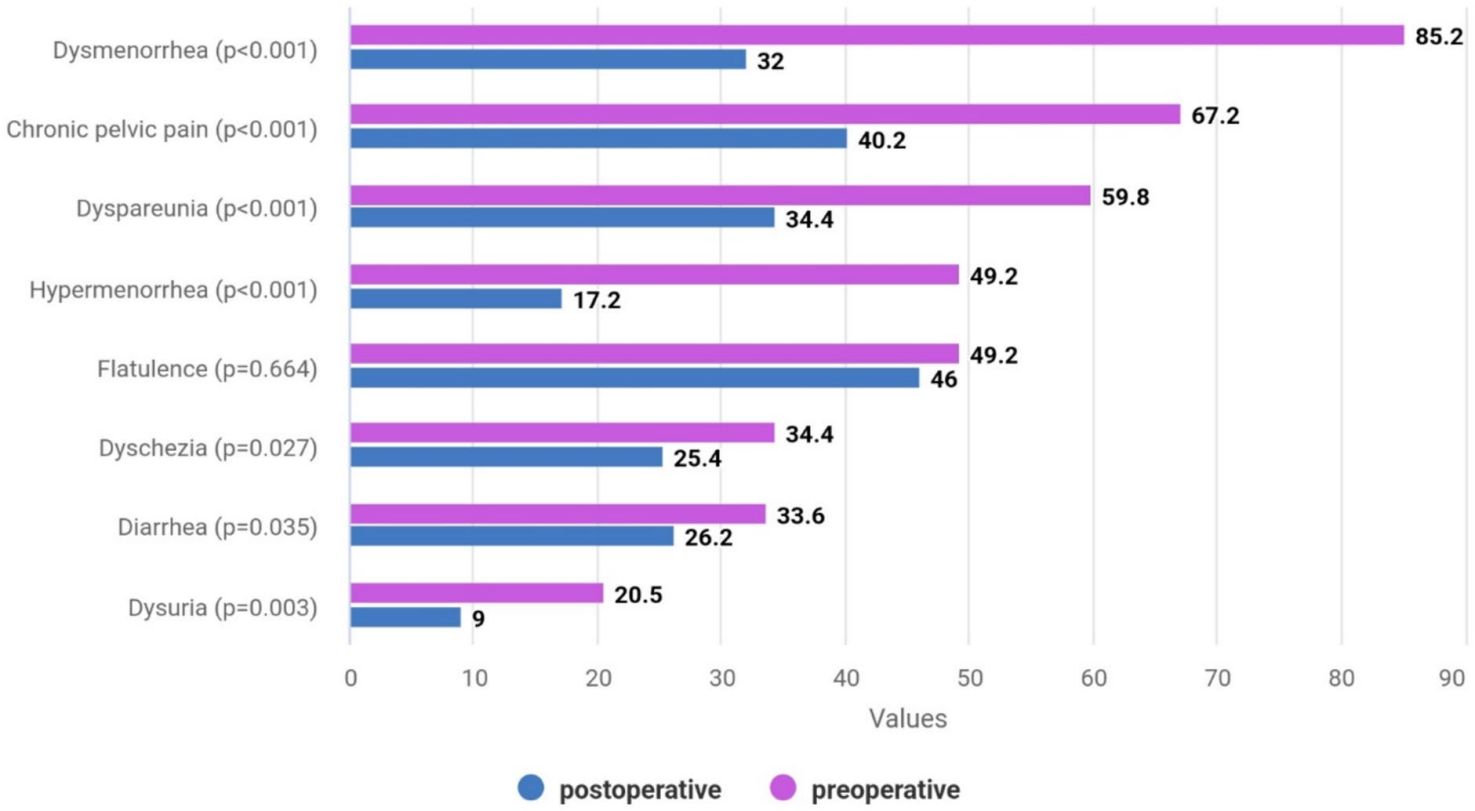
Proportion of women (%) indicating the respective symptom before and after endometriosis surgery

The most frequent clinical symptoms before surgery were menstrual pain (85.2%), CPP (67.2%), dyspareunia (59.8%), hypermenorrhea (49.2%) and flatulence (49.2%) (Fig. 1).

The proportion of women reporting symptoms significantly decreased in the postoperative period: 39 patients (32.0%, p > 0.001) still suffered from dysmenorrhea, but 18 of them said that is was less painful. After surgery, 49 (40.2%, p < 0.001) women still had CPP. However, 23 of them reported improvement. For 26 (31.7%) out of 82 patients with pre-operative CPP, surgery had no effect. Pain during intercourse was still present in 42 cases (34.4%, p < 0.001). Postoperatively, flatulence was the most common (46.7%) symptom. Constipation decreased from 40.2% to 31.1% (p = 0.035), pain during defecation decreased from 34.4% to 25.4% (p = 0.027), and diarrhea decreased from 33.6% to 26.2% (p = 0.035).

**Fig. 2:**
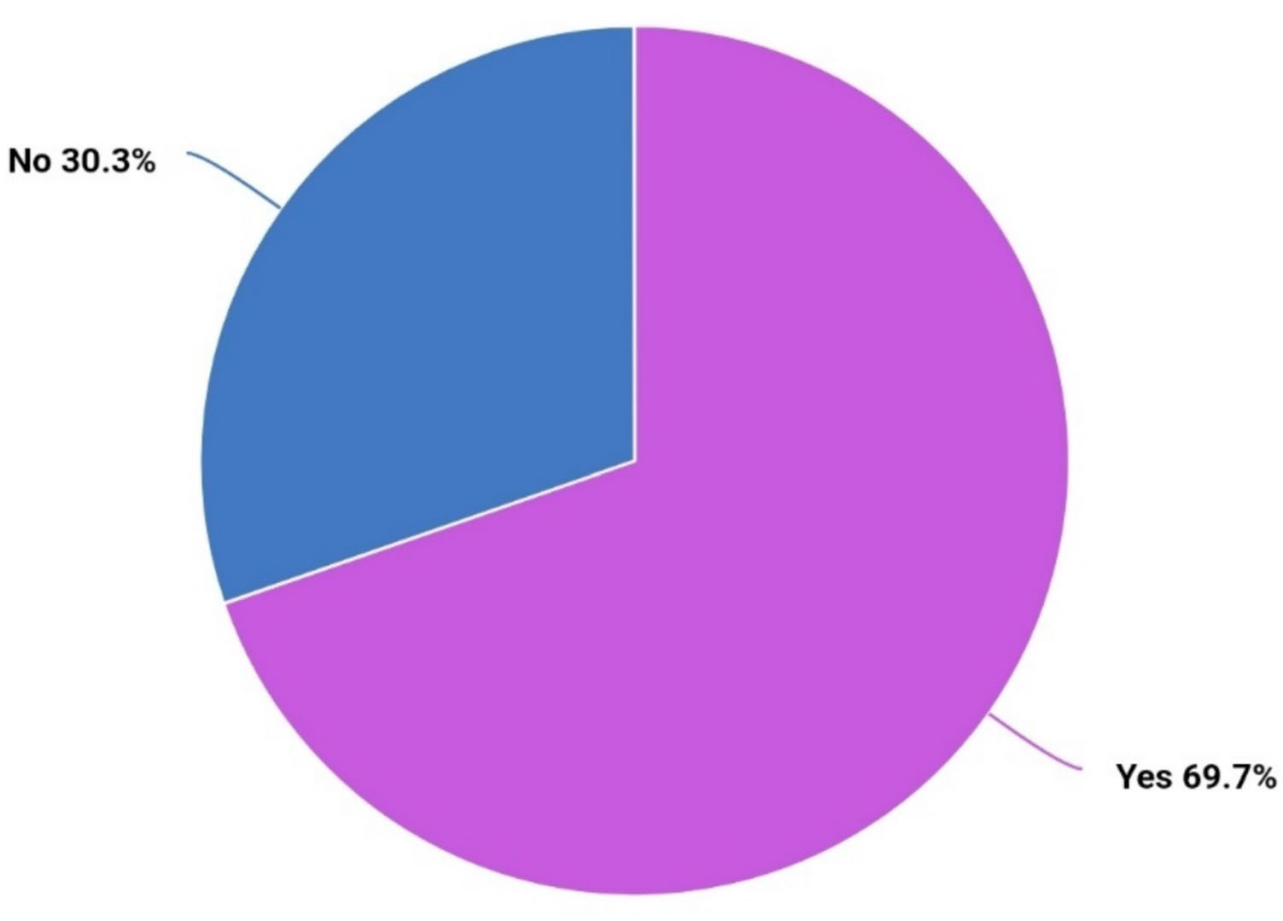
Subjective improvement of general condition after surgery. Proportion of patients who answered “yes” resp. “no” when questioned “Do you think your quality of life has improved after surgery?”

At the time of interview, 69.7% (n = 85) of the patients stated that their subjective general condition had improved compared to the situation before surgery (Fig. 2). The mean SF–12 score in the physical domain was 46.7 (range 17.4–65.9) and 45.7 (range 8.8–62.6) in the mental domain.

Most patients (n = 97, 79.5%) reported that they did not require repeated surgery. Ten women (8.2%) underwent surgery because of recurrence of symptoms and endometriotic lesions within the first year, 8 (6.6%) underwent a second surgery between one and two years after primary surgery, and 7 (5.7%) after more than 2 years. Further details (specific indication, surgical approach, intraoperative findings, etc.) regarding the reoperations were not available.

### Pain intensity and use of analgesics

Patients were asked to score their overall pain intensity on a NRS ranging from zero (no pain) to 100 (highest imaginable pain). There was no further detailed differentiation regarding painful symptoms. Twelve patients (9.8%) stated a pre-operative score of 20 or lower (no or mild pain) and were excluded from the following analysis. None of these patients had a change in NRS Score postoperatively and none used analgesics before or after surgery. Indications for surgery in these patients were as follows: three infertility with suspected endometriosis, three dysmenorrhoea, two suspect adnexal finding, one rectal bleeding, one hypermenorrhoea and one had a combination of dysmenorrhoea, dyschezia and CPP. One patient underwent surgery because of uterus myomatosus and endometriosis was found and resected during surgery.

**Fig. 3:**
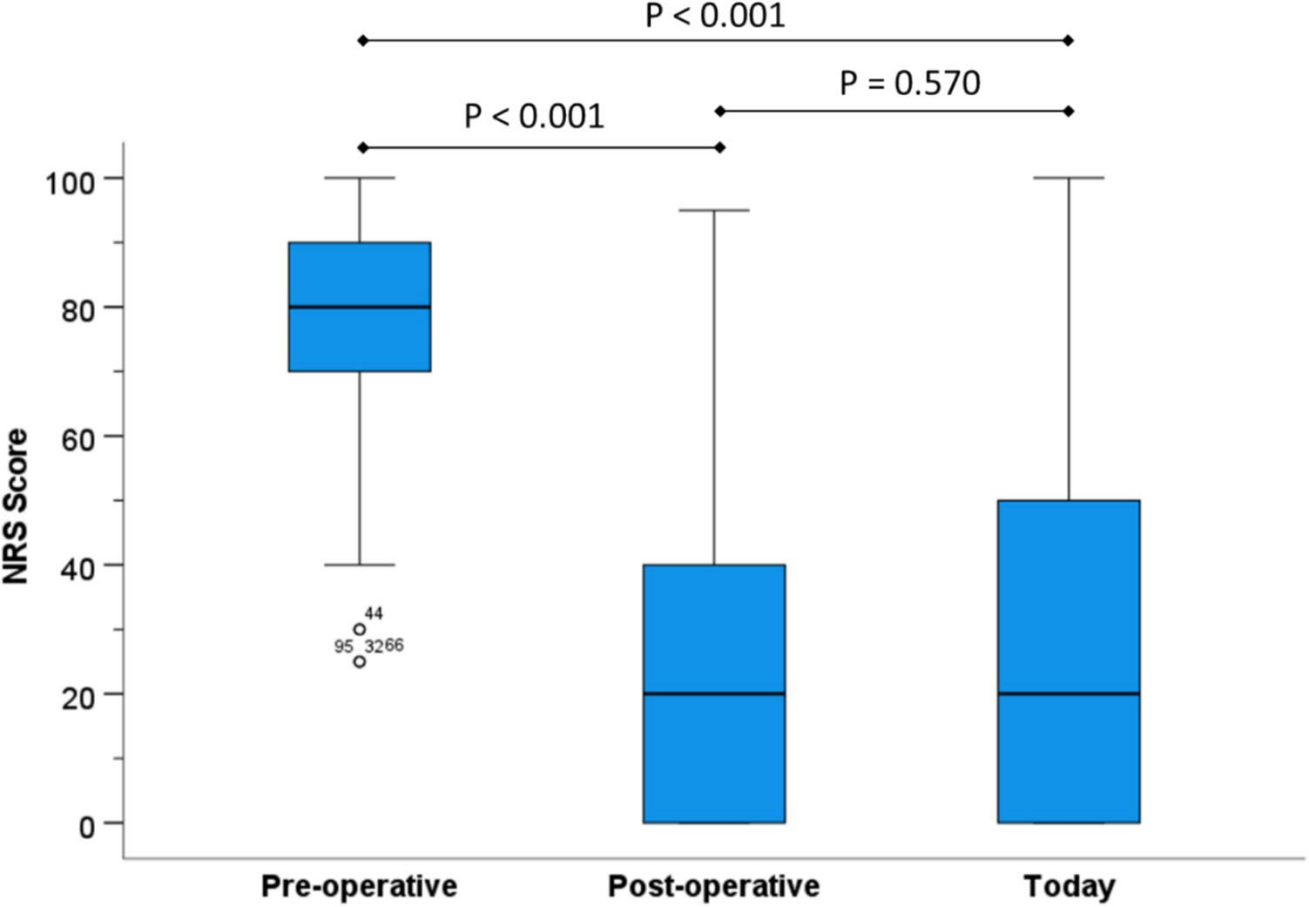
Boxplot of overall pain intensity pre-operatively, post-operatively and at the time of the telephone interview as stated by patients using a numeric rating scale (NRS) ranging from zero (no pain) to 100 (highest imaginable pain). Median time between surgery and interview was 6 years (range 4.3–7.8). Because data showed a non-parametric distribution, the Wilcoxon signed rank-test was applied for statistical analysis

For the remaining patients (n = 110), the mean NRS Score was significantly lower after surgery (26.6 vs. 77.2, p < 0.001, Wilcoxon signed rank-test), corresponding to a change from severe to mild pain. Pain relief was long-lasting, with a mean NRS Score of 28.2 (p = 0.570) and a proportion of 32.7% (n = 36/110) patients with NRS = 0 at the time of questioning (Fig. 3). Twenty-three % (25/110) of the patients claimed that they did not use analgesics, neither pre-operatively nor at time of interview. Of the remaining patients, the majority could reduce consumption of analgesics after surgery (n = 64/85, 75.3%). At the time of the interview, 37.6% (n = 32/85) were still taking analgesics.

In order to analyze whether there are groups of patients, who have a higher chance for pain reduction after endometriosis surgery, univariate ANCOVA analysis was performed (Table 2). Adjusted mean NRS Score at time of interview was significantly inferior in patients with pre-operatively CPP (32.1 ± 3.2, p = 0.017) and women without permanent partnership (44.7 ± 6.9, p = 0.010).

**Table 2:** Overall pain scores pre-operatively and at time of interview (“today”) based on numeric rating scale ranging from zero (no pain) to 100 (highest imaginable pain). Median time between surgery and interview was 6 years (range 4.3–7.8). Adjusted values and corresponding p values were obtained with univariate ANCOVA. Only patients with a pre-operative NRS Score of > 20 were included

Factor		n	NRS pain score			
			Pre-operative Mean (SD)	Today Mean (SD)	^a^Adjusted today Mean (SE)	p value
Whole cohort	–	110	77.2 (17.7)	28.2 (29.0)	–	**< 0.001**^**b**^
Age [years]	<30	31	81.9 (15.2)	35.0 (28.8)	34.7 (5.2)	0.150
	>30	79	75.3 (18.4)	25.5 (28.8)	25.6 (3.3)	
Obesity (BMI > 30)	<30	89	76.3 (18.4)	30.1 (28.9)	30.2 (3.1)	0.142
	>30	21	80.7 (14.5)	20.2 (28.4)	19.8 (6.3)	
Smoker	Yes	61	75.3 (17.1)	29.2 (28.9)	29.4 (3.7)	0.628
	No	49	79.5 (18.4)	26.9 (29.3)	26.7 (4.2)	
Higher education	Yes	40	78.3 (17.8)	31.9 (30.4)	31.8 (4.6)	0.332
	No	70	76.5 (17.8)	26.1 (28.2)	26.1 (3.5)	
Physical acitivity ≥ 1x weekly	Yes	64	78.4 (17.7)	27.0 (27.5)	26.8 (3.6)	0.561
	No	46	75.4 (17.8)	29.9 (31.1)	30.1 (4.3)	
Permanent partnership	Yes	93	77.1 (18.2)	25.2 (27.1)	**25.2 (2.9)**	**0.010**
	No	17	77.7 (14.9)	44.7 (34.1)	**44.7 (6.9)**	
rASRM (Missing = 9)	I	32	79.8 (19.8)	34.7 (29.9)	34.4 (5.2)	0.535
	II	17	75.0 (16.3)	26.8 (25.0)	27.0 (7.1)	
	III	21	76.4 (15.6)	30.7 (33.0)	30.8 (6.4)	
	IV	31	77.1 (18.1)	23.9 (27.3)	23.9 (5.2)	
CPP	Yes	81	78.3 (17.2)	32.2 (30.6)	**32.1 (3.2)**	**0.017**
	No	29	74.1 (19.0)	16.9 (20.5)	**17.1 (5.3)**	
Dysmenorrhoe	Yes	101	77.8 (16.9)	28.6 (29.5)	28.5 (2.9)	0.702
	No	9	70.6 (25.3)	23.9 (22.9)	24.6 (28.5)	
Dispareunia	Yes	70	79.8 (16.5)	28.3 (27.9)	28.0 (3.5)	0.925
	No	40	72.5 (19.0)	28.0 (31.1)	28.5 (4.7)	
Infertility	Yes	59	75.6 (18.7)	28.3 (27.4)	28.5 (3.8)	0.908
	No	51	79.0 (16.4)	28.0 (31.0)	27.8 (4.1)	
Postoperative hormonal therapy (Missing = 8)	No	40	65.9 (20.6)	23.8 (24.9)	23.8 (5.0)	0.515
	^c^Comb. or estradiol	17	84.0 (13.3)	31.8 (34.4)	34.1 (7.2)	
	^d^Progestin	45	84.1 (11.6)	30.0 (29.9)	29.5 (4.5)	

*CPP* chronic pelvic pain. Bold numbers indicate statistical significance (p ≤ 0.05)

^a^Adjusted for pre-operative NRS-value and the respective factor

^b^Wilcoxon signed rank-test

^c^Combined oral contraceptives or estradiol (two women received estradiol alone for HRT after bilateral adnexectomy)

^d^Oral progestin or hormonal intrauterine device

### Fertility and obstetric outcome

Before the operation, 50.8% of patients with endometriosis (n = 62/122) could not give birth to a child. Of them, 56.5% (n = 35/62) applied for reproductive medical care.

Almost half of the pre-operatively infertile women (n = 28/62, 45.2%) had one or more successful pregnancies after surgical therapy and 40 children were born, including two twins. Information regarding parity after surgery was missing in five patients (8.1%). Nineteen patients (30.6%) reported one or more miscarriages, however it was not indicated whether these occurred before or after surgery. In women with infertility younger than 40 years (n = 49), almost two third (63.2%, n = 28) gave birth after surgery. Vaginal delivery was possible in 50% (19/38) of births. Elective cesarean section was conducted in 39.5% (15/38) and emergency C-section was necessary in 2.6% (1/38). In three cases, no information was given regarding mode of delivery.

A study of the interval between surgery and pregnancy revealed that 35.7% (10/28) of patients conceived within 1 year, 46.4% (13/28) between 1 and 3 years and 5 patients (17.9%) after more than 3 years. Reproductive medicine assistance was demanded by 43.5% (27/62) of women after surgery and by 53.4% (15/28) of women with successful pregnancies.

Complications during pregnancy occurred in 36.8% (14/38): placenta previa 3x, gestational diabetes 3x, velamentous cord insertion 2x, leg edema 2x, and each one case of amnion rupture with infection, prepartum bleeding, premature labor, as well as need for progesterone/prednisolone in first trimester. In three cases, no information concerning course of pregnancy was available.

### Identification of non-responders

As outlined above, surgical treatment of endometriosis was successful for many patients, leading to pain relief, improved subjective general condition, and in some cases, to restoration of fertility.

However, there were patients exhibiting resistance to therapy. No permanent pain reduction was assumed if individual improvement of NRS Score at time of interview was lower than 20 points compared to the pre-operative value. This was the case for 25.5% of patients (28/110). Of these patients, nine (32.1%) still had moderate pain (NRS 25–50), eight (28.6%) had severe pain (NRS 60–70) and 11 (39.3%) had most severe pain (NRS > 70).

Thirty-seven of 122 patients (30.3%) claimed that their subjective general condition had not improved, 25/122 (20.5%) had to undergo reoperation because of recurrence of endometriosis and 29/62 (46.8%) did not gain fertility.

### Failure of pain relief is associated with compromised subjective general condition and increased risk for early reoperation

**Fig. 4:**
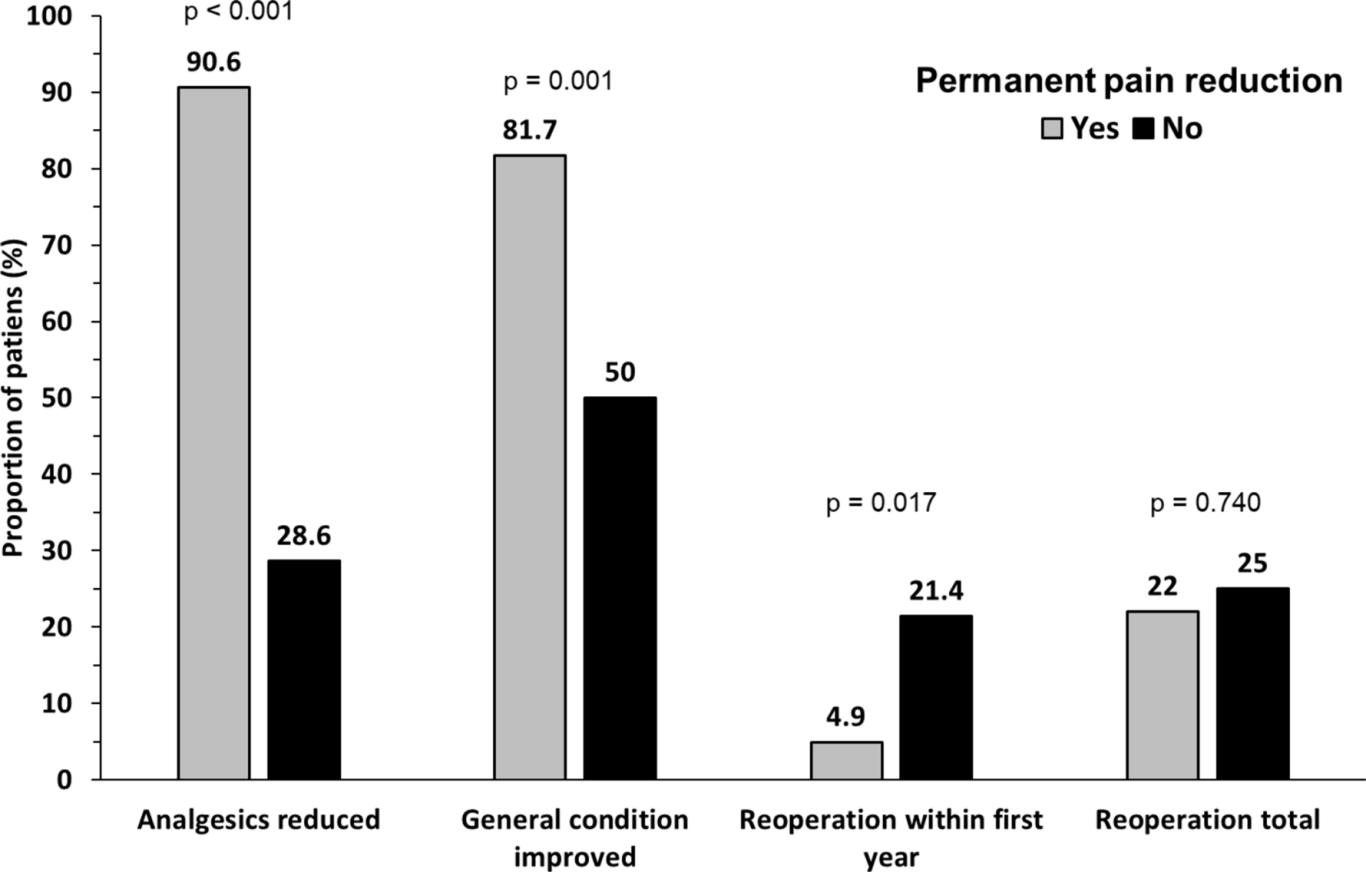
Percentage of patients with the respective outcome depending on achievement of permanent pain reduction. *p* values according to Chi-squared test, except for reoperation within first year, where Fisher’s exact test was applied

Further analysis revealed an association of pain relief with other dimensions of disease burden. If considerable pain reduction was not achieved, a significantly lower proportion of patients reported reduction of analgesics (28.6% vs. 90.6%, p < 0.001) or subjective general condition improvement (50.0% vs. 81.7%, p = 0.001) (Fig. 4). In addition, risk of reoperation within the first year after surgery was increased (21.4% vs. 4.9%, p = 0.017), while total reoperation risk was not different (25.0% vs. 22.0%, p = 0.740).

### Analysis of risk factors for resistance to therapy

To identify pre-operative risk factors for therapy failure, a logistic regression analysis was performed (Table 3). In univariate analysis, risk for failure of pain reduction was increased with younger age (OR per year of age: 0.933, 95% CI 0.876–0.993, p = 0.029), in patients with lower pre-operative pain score (OR per point of pre-operative NRS Score: 0.968, 95% CI 0.944–0.992, p = 0.009) and in patients with CPP (OR 3.869, 95% CI 1.071–13.981, p = 0.039). This was confirmed in multivariate analysis (Table 4). CPP also significantly increased the risk for recurrence, necessitating reoperation (OR in multivariate analysis: 5.191, 95% CI 1.100–24.501, p = 0.038). No predictive factors were identified for therapy resistance in terms of subjective general condition improvement and attainment of fertility.

**Table 3:** Logistic regression analyses for investigation of impact of pre-operative characteristics on risk for failure of treatment. OR (95% CI)

Pre-operative parameter	^a^No permanent pain reduction (n = 28/110, 25.5%)	^b^GC not improved (n = 37/122, 30.3%)	^c^Infertility not overcome (n=29/62, 46.8%)	^d^Reoperation (n=25/122, 20.5%)
Age per year	**0.933 (0.876–0.993)** **p = 0.029**	1.041 (0.989–1.095) p=0.124	**1.169 (1.051–1.301)** **p=0.004**	0.995 (0.940–1.054) p=0.873
Obesity (BMI > 30)	0.637 (0.195–2.085) p = 0.456	0.774 (0.278–2.154) p=0.624	2.652 (0.610–11.519) p=0.193	0.525 (0.143–1.932) p=0.332
Smoking	2.011 (0.814–4.963) p = 0.130	0.624 (0.287–1.358) p=0.235	0.796 (0.278–2.285) p=0.672	1.353 (0.553–3.307) p=0.507
Higher education	1.765 (0.737–4.230) p = 0.202	1.239 (0.555–2.768) p=0.601	0.519 (0.176–1.532) p=0.235	0.872 (0.341–2.228) p=0.775
Physical acitivity ≥ 1x weekly	0.640 (0.270–1.518) p = 0.311	0.747 (0.342–1.629) p=0.463	1.926 (0.653–5.682) p=0.235	2.048 (0.784–5.351) p=0.144
Permanent partnership	0.417 (0.141–1.229) p = 0.113	0.571 (0.199–1.640) p=0.298	0.460 (0.103–2.056) p=0.309	0.565 (0.179–1.786) p=0.331
rASRM-Score I vs. IV	1.796 (0.589–5.471) p=0.303	1.571 (0.524–4.711) p=0.420	0.606 (0.158–2.319) p=0.464	1.080 (0.296–3.946) p=0.907
rASRM-Score II vs. IV	0.735 (0.163–3.308) p=0.688	1.786 (0.519–6.141) p=0.358	0.909 (0.158–2.319) p=0.901	1.687 (0.422–6.743) p=0.459
rASRM-Score III vs. IV	1.071 (0.289–3.973) p=0.918	2.551 (0.794–8.192) p=0.116	1.515 (0.286–8.032) p=0.625	2.224 (0.607–8.144) p=0.228
CPP	**3.869 (1.071**–**13.981)** **p=0.039**	0.724 (0.322–1.626) p=0.434	1.235 (0.410–3.720) p=0.708	**7.407 (1.651**–**33.239)** **p=0.009**
Dysmenorrhoe	1.213 (0.237–6.215) p=0.817	**0.281 (0.100**–**0.784)** **p=0.015**	0.481 (0.081–2.862) p=0.421	1.341 (0.356–5.051) p=0.664
Dispareunia	0.691 (0.288–1.661) p=0.409	**0.440 (0.200**–**0.967)** **p=0.041**	0.909 (0.310–2.669) p=0.862	**3.321 (1.152**–**9.569)** **p=0.026**
Hypermenorrhoe	1.270 (0.535–3.015) p=0.588	0.830 (0.383–1.800) p=0.637	1.635 (0.573–4.660) p=0.358	1.733 (0.709–4.238) 0.228
Infertility	1.472 (0.615–3.525) p=0.386	0.646 (0.297–1.406) p=0.271	–	0.709 (0.293–1.716) p=0.445
^g^COC*	1.500 (0.413–5.450) p=0.538	2.133 (0.671–6.779) p=0.199	–	3.818 (0.936–15.579) p=0.062
^h^Progestin*	0.857 (0.314–2.337) p=0.763	0.731 (0.298–1.795) p=0.495	–	2.567 (0.815–8.082) p=0.107
pre-operative NRS pain score per point	**0.968 (0.944–0.992)** **p=0.009**	**0.978 (0.965–0.992)** **p = 0.002**	1.002 (0.981–1.024) p = 0.829	**1.041 (1.011**–**1.072)** **p = 0.008**

*CPP* chronic pelvic pain. Bold numbers indicate statistical significance (p ≤ 0.05)

^a^Failure in permanent pain reduction was assumed if individual improvement of NRS Score at time of interview was lower than 20 points compared to pre-operative value. Median time between surgery and interview was 6 years (range 4.3–7.8). Only patients with a pre-operative NRS Score of > 20 were included

^b^Patients who answered “no” when questioned “Do you think your quality of life has improved after surgery?”, *GC* subjective general condition

^c^No live birth after endometriosis surgery in patients with infertility before treatment

^d^Need for repeated endometriosis surgery

^g^Combined oral contraceptives

^h^Oral progestin or hormonal intrauterine device

^*^two patients were excluded who had bilateral adnexectomy and subsequent HRT with pure estradiol

**Table 4:** Multivariate logistic regression analyses for investigation of impact of pre-operative characteristics on risk for failure of treatment

OR (95% CI) p value	^a^No permanent pain reduction (n = 28/110)	^b^GC not improved (n = 37/122)	^c^Reoperation (n=25/122)
Age per year	**0.912 (0.850–0.980)** **p = 0.012**	–	–
CPP	**5.544 (1.338–22.965)** **p = 0.018**	–	**5.191 (1.100–24.501)** **p = 0.038**
Dysmenorrhoe	–	0.239 (0.055–1.034) p = 0.056	–
Dispareunia	–	0.697 (0.277–1.753) p = 0.443	2.400 (0.786–7.335) p=0.124
Pre-operative NRS pain score per point	**0.925 (0.944–0.982)** **p = 0.002**	0.985 (0.961–1.010) p = 0.228	1.034 (1.000–1.070) p = 0.053

*CPP* chronic pelvic pain. Bold numbers indicate statistical significance (p ≤ 0.05)

^a^No permanent pain reduction was assumed if individual improvement of NRS Score at time of interview was lower than 20 points compared to pre-operative value. Median time between surgery and interview was 6 years (range 4.3–7.8). Only patients with a pre-operative NRS Score of > 20 were included

^b^Patients who answered “no” when questioned “Do you think your quality of life has improved after surgery?”, *GC* subjective general condition

^c^Need for repeated endometriosis surgery

### Exploring accumulating risk for persisting pain

**Fig. 5:**
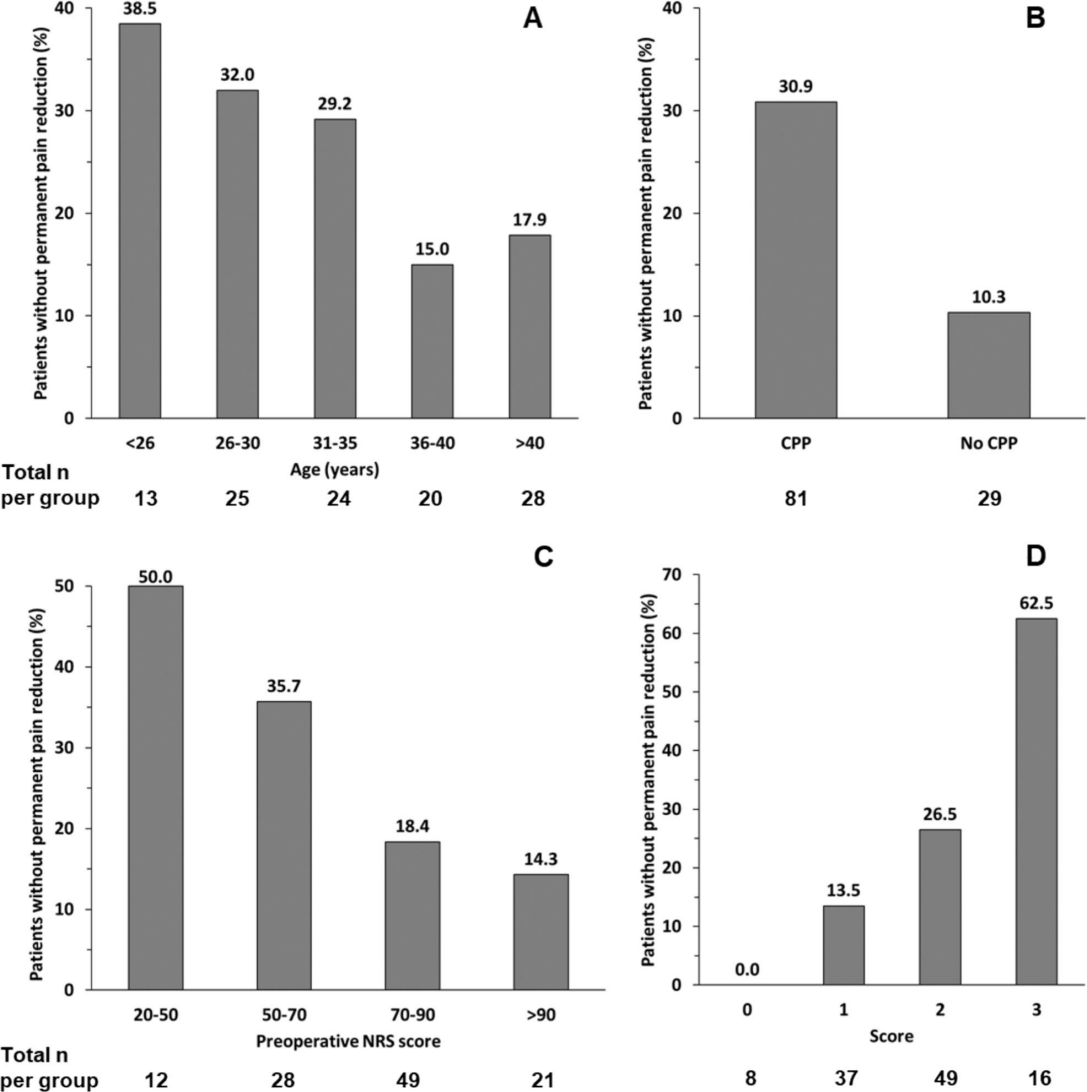
Percentage of patients who do not show permanent pain reduction, depending on age (**A**), chronic pelvic pain (**B**), pre-operative NRS Score (**C**) and a score (**D**) summing up the number of factors depicted in **A–C**, which were present in each patient. NRS, numeric rating scale for pain intensity ranging from zero (no pain) to 100 (highest imaginable pain). No permanent pain reduction was assumed if individual improvement of NRS Score at time of interview was lower than 20 points compared to pre-operative value. Median time between surgery and interview was 6 years (range 4.3–7.8). Only patients with a pre-operative NRS Score of > 20 were included. NRS 20–50 corresponds to mild or moderate pain, NRS 50–70 corresponds to severe pain, NRS 70–90 corresponds to highly severe pain and NRS >90 corresponds to most severe pain

Because younger age, presence of CPP before endometriosis surgery and lower pre-operative NRS Score were associated with a higher risk for persistence of pain, a detailed analysis of these factors was conducted (Fig. 5).

In 29.2–38.5% of patients younger than 36 years, pain was still present at time of interview. In contrast, this was the case in only 15.0–17.9% of patients at age > 35 years (Fig. 5A).

Of patients with CPP prior to surgery, 30.9% had persistent pain in contrast to 10.3% without CPP (Fig. 5B).

Regarding pre-operative pain intensity, the percentage of patients who had no permanent pain reduction was higher in patients with NRS ≤ 70 (35.7–50% vs. 14.3–18.4%, Fig. 5C).

Based on these observations, a score was developed. One point was given for each of the following factors present in a patient: age ≤ 35, CPP before endometriosis surgery and pre-operative NRS ≤ 70. In our cohort, 16 patients had all three factors present at the same time, giving a score of three. These patients had a 62.5% risk for failure of pain reduction (Fig. 5D) after surgery. In contrast, patients who exhibited none or only one of these characteristics had a risk <13.5%.

## Discussion

### Main findings

In this work, patient reported long-term outcome after laparoscopic endometriosis surgery was analyzed in correlation with pre-operative patient and disease characteristics. Most patients reported improvement of main symptoms, subjective general condition as well as fertility, confirming the precision surgery approach as a valuable component of endometriosis treatment.

However, 20.5–30.3% of patients did not respond to therapy in one of the studied domains. Failure in efficient pain reduction was associated with compromised subjective general condition, increased risk for reoperation within the first year after surgery and hindering of analgesics reduction. Utilizing comprehensive statistical analysis, CPP before endometriosis surgery was identified as a strong risk factor for failure in long-lasting pain reduction and recurrence of endometriosis in terms of need for reoperation.

Furthermore, efficient and long-lasting pain reduction was more likely in patients with higher pre-operative NRS scores and with increasing age. Young patients with moderate pre-operative NRS and CPP had a high risk of therapy failure. Postoperative hormonal therapy did not show significant impact in any of the presented analyses. Taken together, our results indicate that it might be possible to identify non-responders prior to surgical endometriosis therapy to route these patients to more effective multidisciplinary treatment regimens [5, 25, 41].

### Interpretation

Proportion of non-responders in endometriosis treatment lies within the range of 13% [42] to 37.5% [33], matching with the observation in our study. Defining a set of factors enabling pre-operative identification of patients with low chance of pain and/or quality of life improvement after surgery is of high importance in order to avoid unnecessary complications and shorten the time to effective treatment [5]. Prospectively, artificial intelligence algorithms could integrate available information and improve decision making. Identification of molecular markers for endometriosis and probability of treatment success would considerably improve situation of patients.

There is indication that patients with higher pre-operative pain intensity may have a higher chance for significant alleviation of pain [42] and increased quality of life [43]. These results are confirmed in our study with regard to pain relief, but not concerning quality of life. Intriguingly, average SF-12 scores in our cohort at the time of interview were comparable to the general population regarding physical health (46.7 vs. 47.3) and only moderately reduced regarding mental health (45.7 vs. 50.3) [44]. This indicates effectiveness of the applied therapy regimen for the majority of patients.

Higher age appears to be associated with better improvement of quality of life [45] and lower risk for recurrence [46, 47]. Our findings support the notion that increasing age is associated with better long-term pain relief in patients undergoing surgery for endometriosis. Older patients, particularly those over 35 years, reported more significant and sustained alleviation of pain, which is linked to age-related changes in pain processing or disease dynamics [20, 21]. In contrast, younger patients, especially those with lower pre-operative pain scores and CPP, exhibited a higher risk of therapy resistance and reoperation. These results underscore the importance of considering age as a factor in treatment planning and highlight the need for tailored, multimodal approaches for younger patients to enhance treatment efficacy.

CPP prior to endometriosis surgery was revealed as a significant risk factor for resistance to therapy in this study. Pre-operative CPP as a predictive factor for recurrence of pain and endometric lesions was also described by other researchers [23, 47]. This poses several consequences for diagnosis and therapy of endometriosis. For patients with CPP a comprehensive therapy regimen is essential, including early interdisciplinary pain treatment approaches with multimodal treatment strategies, which include physiotherapy and osteotherapy, consultation with nutritionist and in more complex cases inpatient psychotherapy and psychosomatic treatment [41, 48]. Focusing solely on the specific disease, while leaving other co-existing conditions unaddressed, often leads to treatment failures, frustration for both patients and providers and lack of improvement. A bio-psycho-social and transdisciplinary approach ensures a comprehensive care plan tailored to the individual patient and offers a greater chance of improving quality of life for these patients. We are currently developing a program in cooperation with the pain unit of the Jena University Hospital. Patients with pelvic pain will undergo interdisciplinary multimodal therapy before endometriosis surgery in order to enhance treatment outcome. Extensive local inflammation, neuronal pain sensitization mechanisms and myofascial dysfunction might play a considerable role in endometriosis-associated CPP [49–51], promoting persistence of pain even after surgical removal of endometriotic lesions. The Central Sensitization Inventory Score (CSI), developed by a Canadian group [24], could be helpful to identify patients with risk for persisting pain after surgical removal of endometriosis. Psychosocial aspects likely also influence the course of pain sensation. This is supported by one observation in this study, showing that women without permanent partnership have lower chance of permanent pain reduction. Definitively, research on mechanisms of pathogenesis of CPP and its treatment should be intensified. Furthermore, gynecologists should be trained in early identification of patients with CPP and/or endometriosis in order to shorten the time to diagnosis and effective treatment. The delay in diagnosis of endometriosis and inefficient treatment might promote development of chronic pain.

In a 2020 study examining fertility in a representative population (n = 1267), pregnancies after endometriosis surgery were reported in 569 patients (44.91%) and live births were 566 (77.2%) [52]. In our investigation, 45.2% of pre-operatively infertile patients had live births after endometriosis surgery. It should be noted that 53.4% of these successful pregnancies occurred after reproductive medical assistance, therefore, it is difficult to determine whether pregnancy developed as a result of assisted reproductive treatment or spontaneously. Efficiency of surgical therapy for improvement of fertility has been shown for minimal [53, 54] as well as deep infiltrating endometriosis [30], although there is only limited evidence from RCTs. In our analysis we could not identify pre-operative parameters predicting the probability of restoration of fertility after endometriosis treatment. Applicability of our data for investigation of this question might have been compromised by a lack of detailed information regarding the attempt to get pregnant after surgery and the sequence of surgery and reproductive medical assistance for each patient.

### Strengths and limitations

This analysis contributes data for identification of pre-operative factors associated with non-responders to surgical endometriosis therapy based on patient reported long-term outcome. However, the study is limited due to its retrospective nature. Questioning of patients was limited to one time point. Consequently, pre-operative NRS scores and symptoms rely on patient’s ability to properly remember the situation prior to surgery. However, there is evidence that pain recall in women with endometriosis is relatively accurate [55]. Furthermore, the retrospectively assessed pre-operative pain intensity scores in our study are comparable to those directly sampled in other studies [33, 41, 56]. We did not complement the NRS by other questionnaires and indices that can provide a more comprehensive evaluation of quality of life and prognosis.

Furthermore, location of endometriosis, ENZIAN classification and comorbidities of patients were not included in the analysis. Because of a response rate of 30%, a selection bias cannot be excluded yet might be low because unavailability of patients was the main reason.

Assessment of change in subjective general condition was limited to one question (“Do you think your quality of life has improved after surgery?”) with only binary answer categories (“yes” resp. “no”).

Data on fertility outcome are limited because we have no information about pre-operative AMH levels, assisted reproductive techniques utilized and the extent of adenomyosis.

Our intention was to investigate long-term outcome of endometriosis surgery. Therefore, we selected a patient cohort that was treated several years ago. Therapy of endometriosis has evolved during the past years. In contrast to our historical cohort, postoperative hormonal therapy is nowadays prescribed for most patients. This could potentially lead to a decrease in the number of non-responders. However, our data do not indicate an association of postoperative hormonal treatment with long-term success of surgical endometriosis therapy.

## Conclusion

Based on our patient-reported outcome study, precision-based surgical endometriosis therapy results in significant and sustained alleviation of disease symptoms for most patients. However, like findings by other researchers, a considerable proportion of patients do not adequately respond to therapeutic intervention. Younger age, lower pre-operative pain intensity, and the presence of chronic pelvic pain (CPP) might represent risk factors for a lack of response. Consequently, reducing the time to diagnosis and initiating effective treatment for endometriosis is crucial to prevent chronification of pain. Identifying patients with a low likelihood of significant pain relief prior to surgery could help optimize individualized treatment regimens including multimodal and interdisciplinary pain management strategies. Our analysis provides a starting point for the design of prospective studies which further characterize the clinical and molecular factors associated with non-responsiveness to surgical endometriosis therapy as a prerequisite for optimization of therapy stratification algorithms.

## Supplementary Information

Below is the link to the electronic supplementary material.

Supplementary file1 (JPG 164 KB)

## Data Availability

Data are available from the authors upon reasonable request.
