# Supplementary figures and images for: Precision surgery for endometriosis: preventing chronic pelvic pain in patients with higher pre-operative pain scores and in patients of advanced age
Source: Arch Gynecol Obstet. 2025 Mar 26;311(4):1111–25. doi: 10.1007/s00404-025-07996-7 (PMC11985621; doi:10.1007/s00404-025-07996-7)

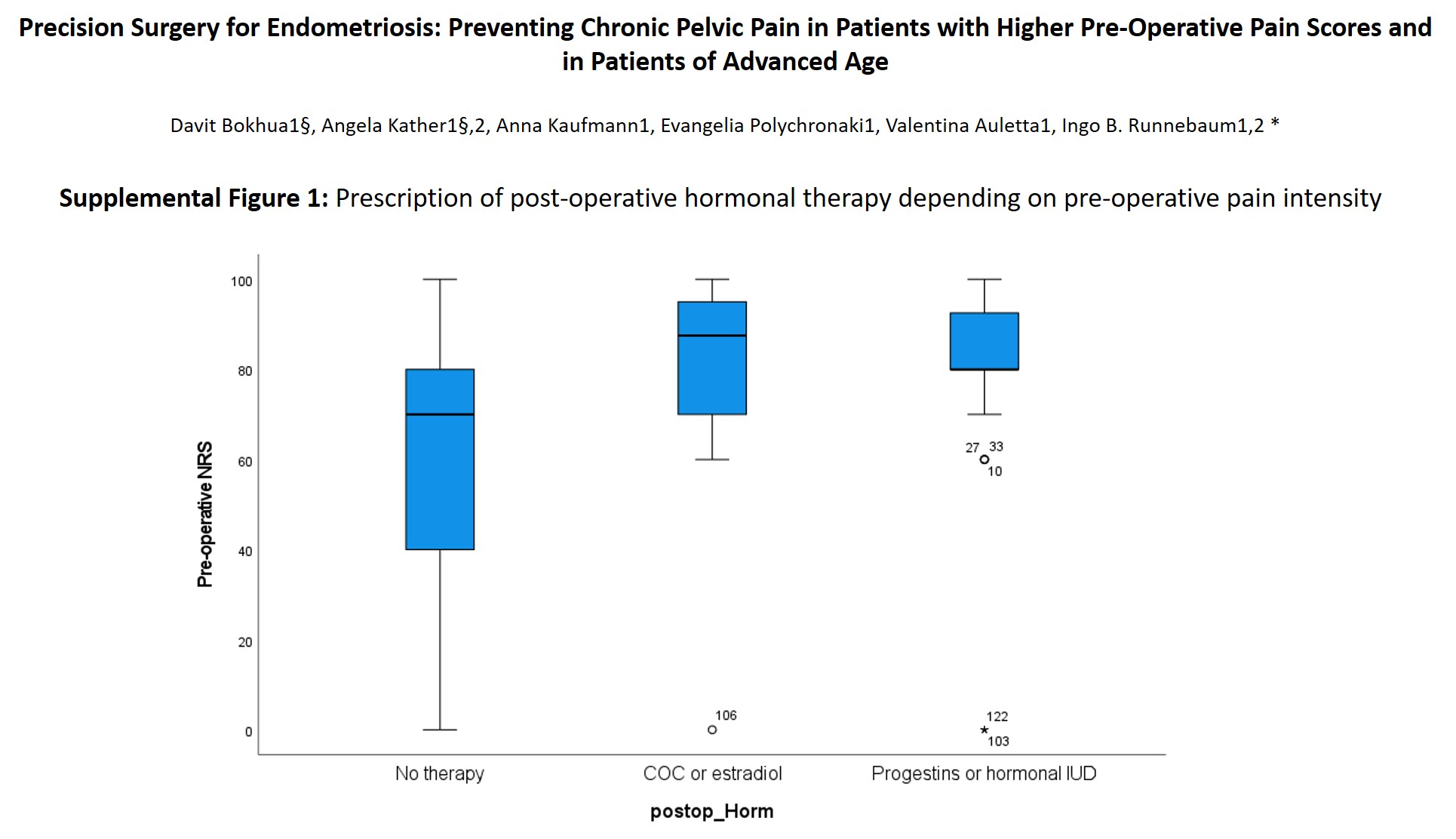

Supplement: Supplementary file 1 — Supplementary file1 (JPG 164 KB) [file 404_2025_7996_MOESM1_ESM.jpg]
